# Independent Evaluation of Rootstock Resistance and Endophytic Bacteria for Managing Citrus Nematode

**DOI:** 10.2478/jofnem-2025-0064

**Published:** 2026-01-31

**Authors:** Seyedeh Najmeh Banihashemian, Seyedeh Negin Mirghasemi, Ali Seraji, Hadi Karimipour Fard, Salar Jamali

**Affiliations:** Citrus and Subtropical Fruits Research Center, Horticultural Science Research Institute, Agricultural Research Education and Extension Organization (AREEO), Ramsar, Iran; Plant Protection Department, Faculty of Agricultural Sciences, University of Guilan, Rasht, Iran; Tea Research Center, Horticultural Science Research Institute, Agricultural Research Education and Extension Organization (AREEO), Lahijan, Iran; Plant Protection Research Department, Kohgiluyeh and Boyerahmad Agricultural and Natural Resources Research and Education Center, AREEO, Yasouj, Iran

**Keywords:** citrus nematode, ecofriendly, integrated management, sustainable agriculture

## Abstract

*Tylenchulus semipenetrans*, a destructive root-parasitic nematode, causes slow decline and significant yield losses worldwide in citrus. Developing resistant cultivars/rootstocks remains an important strategy for the sustainable management of plant-parasitic nematodes in integrated pest management (IPM) systems. Controlling the citrus nematode is challenging due to its wide host range and high adaptability. Although commonly used, chemical nematicides increase production costs and pose risks to human health and the environment. In this study, we separately evaluated the response of five commonly used citrus rootstocks, *Citrus paradisi* (M.) × *Poncirus trifoliata* (L.) Raf. (Citromelo), *P. trifoliata* (L.) Raf. (Poncirus), *Citrus jambhiri* Lush (Rough lemon), *C. volkameriana* Ten. & Pasq. (Volkamer lemon), and *Citrus aurantium* L. (Sour orange), to *T. semipenetrans* infection. In parallel, we evaluated the biocontrol potential of two bacterial strains, *Bacillus safensis* Q.en and *Pseudomonas chlororaphis* P.en, on the susceptible ‘Sour Orange’. The results showed that *T. semipenetrans* exhibited the highest reproduction on ‘Volkamer lemon’ (275 females/g root and 1,150 second-stage juveniles [J2s]/200 g soil), whereas ‘Citromelo’ showed the strongest resistance (8 females/g root and 220 J2s/200 g soil). Application of the bacterial agents to ‘Sour Orange’ significantly reduced the number of females on roots and juveniles in the soil, while also improving plant growth parameters compared to untreated controls. Our findings demonstrate the individual potential of using resistant rootstocks or biocontrol agents as economical, effective, and environmentally safe components for managing *T. semipenetrans*. The resistant genotypes identified may also be useful in future breeding programs.

According to the Food and Agriculture Organization (FAOSTAT, 2023), global citrus (*Citrus* spp., family *Rutaceae*) production and harvested area were 13.4 million tons and 1.46 million hectares, respectively. Iran ranked fourth globally, with 633,000 tons of production and 23,700 hectares of harvested area. In the northern and southern provinces of Iran, citrus is a major horticultural crop. Commonly used rootstocks include Citromelo, Poncirus, Sour orange, Bakraei, and Rough lemon (Mohammad Alian et al., 2018).

As a nutritionally valuable and economically important fruit, citrus contributes significantly to both domestic consumption and export markets in Iran. However, infections caused by plant-parasitic nematodes (PPNs), particularly *T. semipenetrans*, are among the primary causes of citrus orchard decline. The impact of this soil-borne nematode depends on several factors, including tree age, rootstock susceptibility, and nematode population density (Verdejo-Lucas and McKenry, 2004; Banihashemian et al., 2023a). Additional stresses, such as water shortage, poor tree vigor, or *Phytophthora* infections, can exacerbate nematode damage (Jaouad et al., 2020). *T. semipenetrans* has been recognized as a major constraint in citrus production, significantly reducing yield and fruit quality worldwide.

Managing *T. semipenetrans* is challenging due to its wide host range within Citrus, adaptability, and persistence in soil. Although chemical nematicides provide fast suppression, they often harm non-target soil microorganisms and pose environmental and health risks. Consequently, sustainable alternatives for citrus, especially the use of resistant rootstocks and biological control, have gained increasing attention. The efficacy of such integrated strategies has been demonstrated against other nematodes in perennial crops, highlighting their potential for citrus nematode control (Banihashemian et al., 2023c).

Considerable research has focused on the use of PPN-resistant cultivars or rootstocks, particularly in woody perennials (Claverie et al., 2011). For example, nematode-resistant rootstocks have proven effective in managing root-knot nematodes (*Meloidogyne* spp.) in perennial crops, such as kiwifruit (Banihashemian et al., 2023c). Evaluating host responses to *T. semipenetrans* infection represents a critical step in identifying and selecting resistant genotypes. In citrus, choosing a resistant rootstock may serve as a sustainable management strategy against this nematode, though current studies in this area remain limited (Zoubi et al., 2024).

In citrus, Swingle citrumelo and *P. trifoliata* have been reported as resistant rootstocks (Ling et al., 2000; Mohammad Alian et al., 2018). Separately, *C. aurantium* L. (Sour orange) is widely used across Mediterranean regions due to its broad adaptability and high graft compatibility with commercial scions (Beniken et al., 2011; Bowman et al., 2021). However, this rootstock presents significant susceptibility to both *Citrus Tristeza Virus* (CTV) and *T. semipenetrans* nematode infestations (Mohammad Alian et al., 2018; Bowman et al., 2021; Ibrahim et al., 2022). Thus, further research for sustainable management of this nematode, such as developing nematode-resistant rootstock alternatives and biological control with bio-agent antagonists, is necessary. Biological control agents represent a promising option, particularly when resistant genotypes are unavailable. When integrated with resistant cultivars and other agronomic practices, this approach can contribute to long-term nematode suppression.

In sustainable agriculture, beneficial microbes serve as an eco-friendly and good alternative to chemical materials. Endophytic bacteria, for instance, improve plant performance and pathogen resistance via both direct and indirect mechanisms. These mechanisms include improved nutrient uptake, particularly in nitrogen, phosphorus, and iron-deficient soils, by solubilizing minerals and synthesizing phytohormones (auxins, gibberellins, cytokinins, ethylene, and abscisic acid), which simultaneously induce growth and prime plant immune responses, a mechanism directly contributing to pathogen resistance (Rosier et al., 2018; Ali et al., 2021; Banihashemian et al., 2022). Therefore, further elucidating how these microbial functions collectively enhance plant stress adaptation remains a key research objective (Banihashemian and Mirmajlessi, 2025).

There were reports of the successful application of *Bacillus* and *Pseudomonas* species for managing PPNs in woody trees. For example, kiwifruits inoculated with these two bacterial genera exhibited significant reduction of nematode reproduction factors, along with enhanced plant growth parameters, compared to controls (Banihashemian et al., 2023b; Mirghasemi et al., 2025). In addition, *Bacillus* strains exhibited high effectiveness against the *T. semipenetrans*, showing approximately 80% J2 mortality and reduced egg hatching in vitro. These bacteria also enhanced citrus growth and root development (Labiadh et al., 2023). Also, *Pseudomonas* species show significant nematicidal activity against *T. semipenetrans* in citrus under both laboratory and greenhouse conditions (Abdelnabby et al., 2011; Montasser et al., 2012; Hanawi, 2016).

In this study, we first investigated the resistance of five commonly used citrus rootstocks, including Citromelo, Poncirus, Rough Lemon, Volkamer Lemon, and Sour Orange, to the *T. semipenetrans*. Then, the antagonistic potential of two endophytic bacterial strains, namely *B. safensis* Q.en and *P. chlororaphis* P.en, against *T. semipenetrans* was evaluated on the susceptible Sour orange rootstock.

For the sustainable management of *T. semipenetrans*, this study was designed to systematically evaluate the two core components of an integrated strategy in parallel. We hypothesized that (1) commercial citrus rootstocks exhibit varying levels of resistance to the nematode, and (2) endophytic bacteria can effectively reduce nematode damage on a susceptible, widely planted rootstock. Although the direct combination of these tactics remains a key research objective, this study delivers the critical baseline data for each component, confirming their individual potential and facilitating future integration. These two strategies enable the immediate application of the most effective resistant rootstocks in new plantings, while simultaneously providing a biocontrol option for protecting existing orchards established on susceptible rootstocks, such as Sour orange.

## Materials and Methods

### Plant material and growth conditions

Five citrus rootstocks, including *C. paradisi* (M.) × *P. trifoliata* (L.) Raf. (Citromelo), *P. trifoliata* (L.) Raf. (Poncirus), *Citrus jambhiri* Lush (Rough lemon), *C. volkameriana*, Ten. & Pasq. (Volkamer Lemon), and *C. aurantium* L. (Sour Orange), were selected to evaluate their resistance to *T. semipenetrans*. Seeds of each rootstock were surface-sterilized by immersion in 70% (v/v) ethanol for 2 min, followed by three rinses with sterile distilled water. The sterilized seeds were then germinated in trays containing a sterilized mixture of cocopeat, sand, and perlite in a 1:1:1 ratio (v/v/v). The 5-month-old seedlings were transferred to pots (2 L volume) filled with the same sterilized substrate. All plants were grown under greenhouse conditions at 25 ± 2°C and 70% relative humidity, in a controlled environment located in northern Iran. Plants were irrigated regularly and fertilized as needed to ensure uniform growth prior to nematode inoculation.

### Nematode inoculum and identification

To obtain *T. semipenetrans* inoculum for experiments, soil and root samples were collected from nematode-infested citrus orchards in Ramsar city, Mazandaran province, northern Iran. Single egg masses were extracted from infected roots using a stereomicroscope (Nikon SMZ800, Nikon Corporation, Tokyo, Japan). A single egg mass was then inoculated onto the roots of the susceptible Rough lemon seedlings to establish, maintain, and multiply a pure nematode population in a greenhouse at 25 ± 2°C and 70% relative humidity. After 3 months, the roots were assessed for nematode infection. To visualize female nematodes, roots were stained with an acid fuchsin solution (0.39 g acid fuchsin in 75 mL distilled water and 25 mL glacial acetic acid) and then destained using a glycerol–HCl solution (100 mL glycerol + 100 μL HCl).

Second-stage juveniles (J2s) were extracted using the tray method (Whitehead and Hemming, 1965) and stored at 4°C for up to 24 hr until further use. Molecular identification of *T. semipenetrans* was performed by sequencing the D2–D3 expansion segment of the LSU rDNA region, using primers and protocols described by Nunn (1992). The obtained sequence was submitted to GenBank under accession number PV698423.

### Plant growth parameters and nematode assessment

The 5-month-old seedlings of citrus were inoculated with 2,000 J2s of *T. semipenetrans* through four holes around the root zone. Non-inoculated seedlings were used as healthy controls and maintained under identical greenhouse conditions. Plants were irrigated every 7 days based on growth requirements. After 60 days of incubation, seedlings were carefully uprooted. Roots were washed, stained with 0.01% acid fuchsin solution, and destained using a glycerol solution to visualize nematode infection.

The population of *T. semipenetrans* was evaluated by counting the number of female nematodes per gram of root and the number of J2s per 200 g of soil under a stereomicroscope (Nikon SMZ800) (Whitehead and Hemming, 1965). To evaluate the resistance level of citrus rootstocks to *T. semipenetrans*, a nematode female index (NFI) was calculated based on a 0–5 rating scale Table 1.

**Table 1:** Rating scale for evaluating the level of resistance/susceptibility of citrus rootstocks based on the number of female nematodes per gram of root.

**Scale**	**Number of *T. semipenetrans* females/gram of root**	**Resistance rating**
0	0	Immune (I)
1	1–10	Resistant (R)
2	11–30	MR
3	31–60	MS
4	61–100	Susceptible (S)
5	>100	HS

*Note*: The resistance rating scale was adapted from established methods in nematology (e.g., Banihashemian et al., 2023c; Zoubi et al., 2024) and calibrated based on the distribution of nematode population counts observed in this study.

HS, highly susceptible; MR, moderately resistant; MS, moderately susceptible.

In addition, citrus growth parameters, including shoot and root fresh weights, were recorded immediately after harvest. Dry weights were determined after oven-drying the samples at 70°C for 72 hr. All experiments were conducted in a completely randomized design (CRD) with five replicates per treatment, and the entire experiment was repeated twice.

Correlation and regression analyses were performed to assess the relationship between nematode infection and plant growth. The percentage reduction for each growth parameter (shoot/root fresh and dry weight) was calculated for each biological replicate using the formula: Percentage reduction = ([Value of control – Value of infected]/Value of control) × 100. Pearson correlation coefficients were then calculated between the mean number of females per gram of root and the mean percentage reduction values for each rootstock.

### Preparation and characterization of bacterial antagonists

The endophytic bacterial isolates *B. safensis* strain Q.en (GenBank accession OK415424) and *P. chlororaphis* strain P.en (GenBank accession OK415418) was previously isolated from apparently healthy kiwifruit plants in northern Iran (Banihashemian et al., 2022). Isolation of strains Q.en and P.en was performed following the method described by Wicaksono et al. (2018). The isolates were cultured on sucrose nutrient agar (NAS) media and incubated at 25°C for 48 hr to ensure optimal growth. The pure colonies were obtained by successive re-culturing before storage at −80°C in 60% glycerol.

### Inoculation of *B. safensis Q.en* and *P. chlororaphis P.en* on seedlings and evaluation of plant growth parameters

The endophytic bacteria were cultured in liquid Luria-Bertani (LB) medium (yeast extract 5 g, NaCl 10 g, tryptone 10 g, distilled water 950 mL) at 25°C with shaking at 200 rpm for 48 hr to obtain bacterial suspensions. The suspensions were centrifuged at 6,000 rpm for 5 min and washed three times with sterile distilled water. The bacterial pellets were then resuspended in sterile distilled water to reach an optical density (OD_600_) of 0.5.

To evaluate the efficacy of the bacteria against *T. semipenetrans*, 5-month-old Sour orange seedlings were selected for this bioassay. This rootstock was chosen because it is sufficiently susceptible to allow clear evaluation of biocontrol effects, while also being the predominant and traditional rootstock in northern Iranian orchards, making the findings directly relevant to local growers. The seedlings were inoculated with 40 mL of bacterial suspension (10^7^ CFU/mL) of *B. safensis* Q.en and *P. chlororaphis* P.en. After 48 hr, 2,000 J2s of *T. semipenetrans* were inoculated onto the roots of Sour orange seedlings, which were maintained under controlled greenhouse conditions as described above.

Four treatments were considered as follows: (i) control: treated with sterile distilled water only; (ii) inoculated with *T. semipenetrans* only; (iii) pretreated with *B. safensis* strain Q.en followed by *T. semipenetrans* inoculation; and (iv) pretreated with *P. chlororaphis* strain P.en followed by *T. semipenetrans* inoculation. The experiment was conducted in a CRD with five replicates per treatment, and the entire experiment was repeated twice.

### Statistical analysis

Quantitative data were analyzed using SAS software version 9.1 (SAS Institute Inc., Cary, NC, USA) with a one-way analysis of variance (ANOVA). Means ± standard error (X ± SE) was calculated for all experimental data. Differences between treatments were considered significant at *P* < 0.05, based on the least significant difference (LSD) test.

## Results

### Response of citrus rootstocks to *T. semipenetrans*

**Figure 1:**
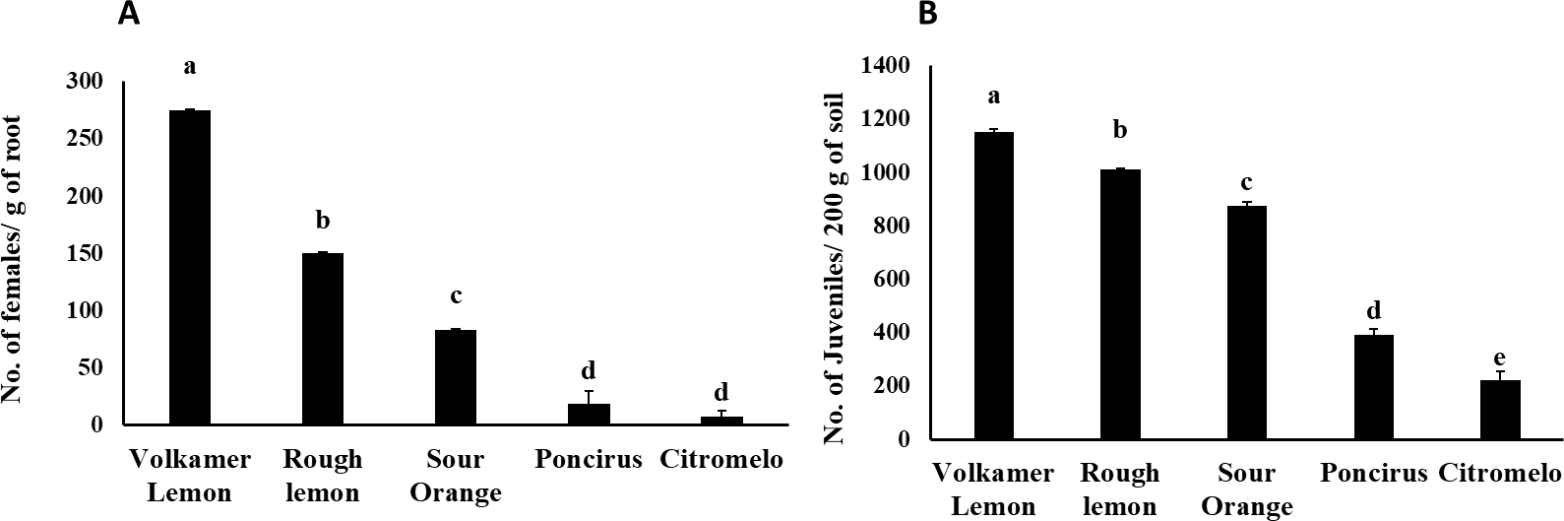
Reaction of citrus rootstocks to infection by *T. semipenetrans*. (A) Number of females on roots; (B) number of J2s in the soil. Different letters denote statistically significant differences (*P* < 0.05). Error bars represent the SEM. SEM, standard error of the mean.

The response of the five citrus rootstocks to infection by *T. semipenetrans* is shown in Figures 1A and 1B. Evaluation of the number of female nematodes that penetrated the roots revealed a significant difference among the rootstocks (*P* < 0.0001). The results showed that the highest number of *T. semipenetrans* females (275/gram of root) was recorded in ‘Volkamer lemon’, whereas the lowest numbers were observed in ‘Citromelo’ and ‘Poncirus’, respectively (Figs. 1A,B).

The rating scale indicated that ‘Volkamer lemon’ and ‘Rough lemon’ were highly susceptible, with >100 female nematodes per gram of root. ‘Sour Orange’ was considered susceptible, with 81–100 females per gram of root. ‘Citromelo’ and ‘Poncirus’ were categorized as resistant and moderately resistant (MR), with 6–10 and 11–30 female nematodes per gram of root, respectively. Notably, ‘Citromelo’ showed significantly greater resistance than ‘Poncirus’ (Table 1; Fig. 1A).

In addition, statistical analysis showed significant differences (*P* < 0.05) in the density of J2s in the soil among the tested rootstocks. The number of J2s per 200 g of soil was significantly higher in ‘Volkamer lemon’ and ‘Rough lemon’, while ‘Citromelo’ and ‘Poncirus’ exhibited significantly lower nematode densities. The lowest J2 count was observed in ‘Citromelo’ (Fig. 1B).

**Figure 2:**
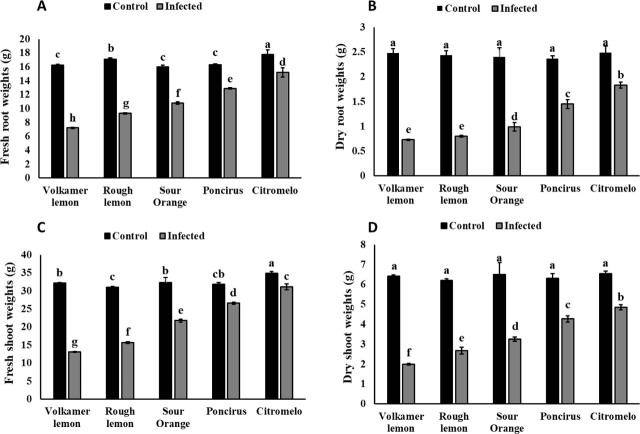
Growth parameters of plants in response to infection by *T. semipenetrans*. (A) Fresh root weight; (B) dry root weight; (C) fresh shoot weight; and (D) dry shoot weight. Different letters indicate statistically significant differences (*P* < 0.05). Error bars represent the SEM. SEM, standard error of the mean.

Moreover, fresh and dry shoot and root weights varied among the rootstocks and were generally reduced in inoculated plants compared to the non-inoculated controls. Root fresh and dry weights were significantly decreased in nematode-infected rootstocks (Figs. 2A,B). The highest reduction in root fresh weight was observed in ‘Volkamer lemon’ (55.66%) and ‘Rough lemon’ (45.65%), followed by ‘Sour orange’ (32.42%). In contrast, the lowest reductions were recorded in ‘Citromelo’ (14.41%) and ‘Poncirus’ (20.91%) (Fig. 2A).

Similarly, ‘Volkamer lemon’ (70.56%), ‘Rough lemon’ (67.21%), and ‘Sour orange’ (58.75%) exhibited the greatest reduction in root dry weight, while ‘Citromelo’ (26.51%) and ‘Poncirus’ (38.82%) showed the least reduction (Fig. 2B).

Shoot fresh and dry weights were significantly reduced in infected rootstocks compared to the control (Figs. 2C,D). The greatest reduction in shoot fresh weight was recorded in ‘Volkamer lemon’ (59.32%), ‘Rough lemon’ (49.44%), and ‘Sour orange’ (32.56%). In contrast, the lowest reduction was observed in ‘Citromelo’ (10.71%) and ‘Poncirus’ (16.31%) (Fig. 2C).

Similarly, shoot dry weight showed the highest reduction in ‘Volkamer lemon’ (68.95%), followed by ‘Rough lemon’ (56.84%) and ‘Sour orange’ (49.92%). The lowest dry weight reduction was observed in ‘Citromelo’ (25.95%) and ‘Poncirus’ (32.22%) (Fig. 2D).

**Figure 3:**
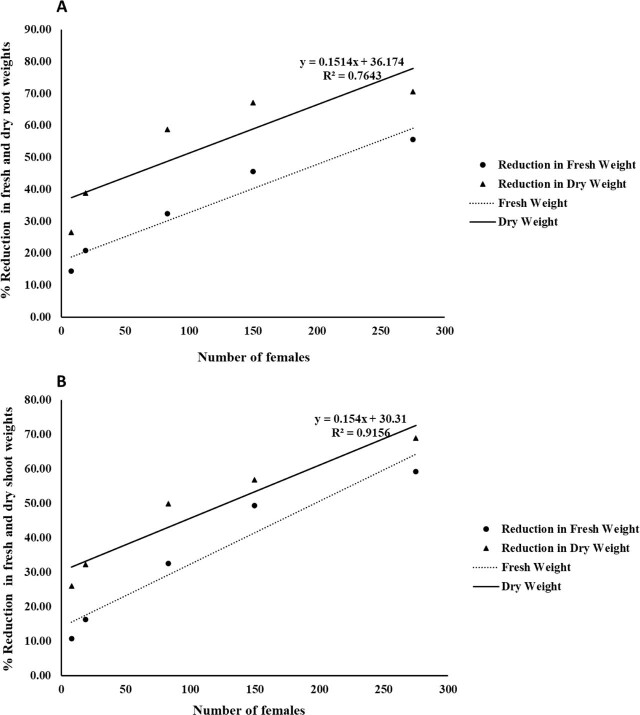
Relationships between the number of *T. semipenetrans* females and percentage of reductions in fresh and dry root (A) and shoot (B) weights of citrus plants. See the key within the figure for symbol and line identifications.

Regression analysis revealed significant positive correlations between the number of *T. semipenetrans* females and reductions in fresh and dry shoot and root weights (Figs. 3A,B).

### Antagonistic effects of the endophytic bacteria *B. safensis Q.en* and *P. chlororaphis P.en against T. semipenetrans* in greenhouse studies

**Figure 4:**
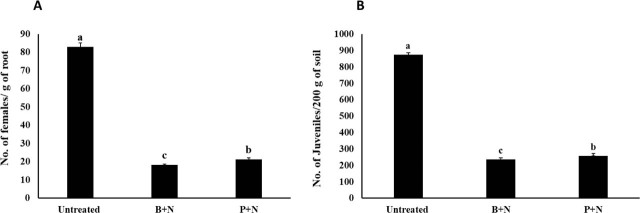
Antagonistic effects of *B. safensis* strain Q.en and *P. chlororaphis* strain P.en against *T. semipenetrans* in citrus under greenhouse conditions. (A) Number of females per gram of root. (B) Number of second-stage juveniles (J2s) per 200 g of soil. Different letters denote significant differences (*P* < 0.05). Data are means of five replicates repeated twice independently (*n* = 10). Error bars represent the SEM. Untreated (nematode-inoculated); B + N = *B. safensis* + Nematode; P + N = *P. chlororaphis* + Nematode. SEM, standard error of the mean.

**Figure 5:**
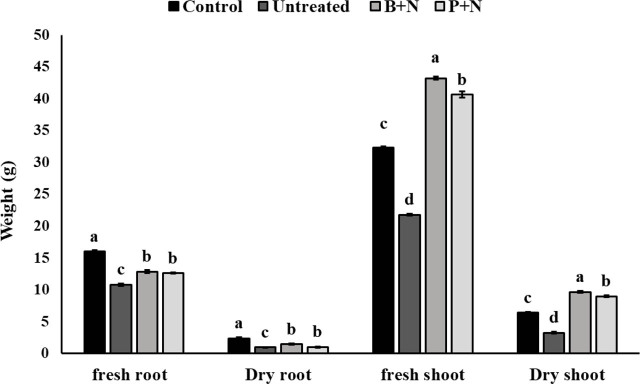
Effects of *B. safensis* strain Q.en and *P. chlororaphis* strain P.en on citrus growth parameters under greenhouse conditions. Different letters denote significant differences (*P* < 0.05). Data are the mean of five replications that were repeated two times independently (*n* = 10). Error bars represent the SEM. Untreated (nematode-inoculated); B + N = *B. safensis* + Nematode; P + N = *P. chlororaphis* + Nematode. SEM, standard error of the mean.

The number of *T. semipenetrans* females in the roots was significantly reduced in citrus seedlings pretreated with *B. safensis* strain Q.en and *P. chlororaphis* strain P.en, compared to the untreated control (Fig. 4). In addition, plant growth parameters, particularly shoot fresh and dry weights, were significantly improved following bacterial inoculation (Fig. 5)

## Discussion

Citrus production faces significant challenges of biotic and abiotic stresses. Among these, the *T. semipenetrans* poses a serious threat to the citrus industry in Iran and worldwide, significantly affecting plant growth and yield (Mohammad Alian et al., 2018; Kumar and Arthurs, 2021).

Given these challenges, biological control agents have gained particular attention as eco-friendly and sustainable strategies for disease management. Integrating resistant rootstocks with microbial antagonists provides a promising approach against nematodes, which are difficult to control.

The use of disease-resistant rootstocks has a long history in citrus production, although their resistance levels to *T. semipenetrans* vary widely among genotypes. In this study, we first evaluated the resistance of five commonly used citrus rootstocks (Citromelo, Poncirus, Sour orange, Volkamer lemon, and Rough Lemon) to nematode infection. Additionally, we investigated the antagonistic effects of two endophytic bacterial strains (*B. safensis* and *P. chlororaphis*) on Sour orange seedlings.

Our findings showed that both bacterial strains significantly improved shoot and root biomass in infected and non-infected plants. In our experiment, *T. semipenetrans* exhibited high reproduction rates on Volkamer lemon and Rough lemon (275 and 150 females/g root, respectively), followed by Sour orange (83.1 females/g root). In contrast, Citromelo and Poncirus supported much lower nematode reproduction (8 females/g root and 19 females/g root, respectively). These results confirm that Citromelo is the most resistant and Poncirus MR among the tested rootstocks.

These findings are consistent with earlier reports demonstrating that the genetic background of rootstocks plays a key role in nematode resistance (Ling et al., 2000; Mohammad Alian et al., 2018; Zoubi et al., 2024). Rootstocks with inherent resistance offer a valuable resource for breeding programs or use in nematode-infested orchards.

Also, nematode infection significantly reduced growth parameters in all rootstocks compared to the non-inoculated controls. The greatest reduction in fresh and dry shoot and root weight was observed in Volkamer lemon and Rough lemon, followed by Sour orange. In contrast, Citromelo and Poncirus exhibited the least reduction, aligning with their resistance levels. While the overall negative correlation between nematode density and plant growth was clear across all rootstocks, future studies could characterize these relationships more precisely at the individual genotype level to better understand host-specific response patterns.

Application of endophytic bacteria to Sour orange seedlings reduced nematode severity and improved plant development, likely by enhancing nutrient uptake and inducing systemic resistance through production of secondary metabolites (Liu et al., 2019; Banihashemian et al., 2023b). Previous studies have similarly shown that endophytic *Bacillus and Pseudomonas* spp. can suppress PPNs and improve host growth across multiple crops (Hu et al., 2018; Tran et al., 2019; Vetrivelkalai, 2019; Banihashemian et al., 2023b).

The significant enhancement of plant growth in pretreated treatments with bacteria is interpreted as a combined effect of direct nematode suppression and the plant growth-promoting capabilities of the endophytic strains. This interpretation is consistent with the established role of endophytic bacteria, including our own prior findings where endophytic bacteria significantly improved plant growth and primed defense responses in the absence of pathogens (Banihashemian et al., 2023c). This supports the conclusion that the observed benefits are derived from the multifaceted action of endophytic bacteria as a management strategy.

The observed suppression of *T. semipenetrans* by *B. safensis* and *P. chlororaphis* is likely mediated through a combination of direct and indirect mechanisms. Directly, these strains may produce a range of bioactive secondary metabolites, such as cyclic lipopeptides or hydrogen cyanide, with documented nematicidal activity that could paralyze or kill J2s before root penetration (Banihashemian et al., 2022; Mabrouk et al., 2025). The nematicidal and growth-promoting effects observed in our study align with recent reports of rhizosphere bacteria efficacy against *T. semipenetrans*, where various *Bacillus* species caused up to 85% J2 mortality, inhibited egg hatching, and enhanced citrus root growth (Labiadh et al., 2023). Similarly, a bacterial microbiome achieved near-complete juvenile mortality (98.98%) and significantly suppressed nematode populations under greenhouse conditions (Zoubi et al., 2025). Indirectly, their well-established capacity to induce systemic resistance (ISR) in host plants could prime the citrus root system, enhancing the production of defense-related compounds upon nematode challenge (Subedi et al., 2020; Banihashemian et al., 2023b). Furthermore, effective root colonization and biofilm formation by these endophytes are crucial prerequisites for both nutrient competition and sustained interaction with the host plant, creating a hostile rhizosphere environment for the nematode.

It is noteworthy that the number of females per gram of root was selected as the key metric in this study because it directly quantifies the parasitic success of *T. semipenetrans* at its most damaging life stage. This provided a uniform measure for comparing both host resistance and biocontrol efficacy.

The application of these promising greenhouse results to field efficacy requires consideration of several factors. The persistence and activity of the inoculated bacterial strains can be influenced by field variables such as soil type, temperature fluctuations, moisture content, and the native microbial community. Formulation of the biocontrol agents into stable products (e.g., solid powders or liquid concentrates) and application methods that protect them from environmental stresses (e.g., through seed coating or drip irrigation) will be critical for success. Supporting this, a recent study demonstrated that liquid formulations of *P. chlororaphis* and *Bacillus velezensis* significantly suppressed root-knot nematode in kiwifruit under both greenhouse and field conditions, highlighting the importance of proper formulation for field efficacy (Mirghasemi et al., 2025). Future field trials should monitor the population dynamics of the applied strains and their functional persistence throughout the growing season to validate their long-term control potential under real-world conditions.

In summary, we demonstrated that Citromelo and Poncirus showed strong and moderate resistance, respectively, to *T. semipenetrans*. We also confirmed the biocontrol efficacy of *Bacillus* and *Pseudomonas* spp. against this nematode in Sour orange seedlings. These findings demonstrate the individual efficacy of both resistant rootstocks and biocontrol agents, providing the key components for an environmentally friendly strategy to manage citrus nematode infestations.

Notably, while Volkamer lemon was the most susceptible rootstock, Rough lemon showed slightly lower susceptibility, aligning with previous reports (Giudice and Inserra, 1980; Ibrahim et al., 2022). The application of *Bacillus* and *Pseudomonas* strains effectively suppressed nematode infection, highlighting their potential as biocontrol agents.

The combined evaluation of rootstock resistance and biocontrol provides a comprehensive framework for nematode management. The identification of Citromelo and Poncirus as highly resistant rootstocks offers a long-term solution for new plantings, while the demonstrated efficacy of the endophytic strains provides an immediate tool for protecting existing orchards on susceptible rootstocks like Sour orange. Most importantly, this parallel evaluation provides the foundation for direct testing of the integration of these resistant rootstocks with biocontrol agents in field conditions.

## Conclusion

These findings offer practical implications for citrus production in nematode-infested regions. Implementing resistant rootstocks (especially Citromelo and Poncirus) or endophytic antagonists can reduce reliance on chemical nematicides and promote sustainable orchard management. Our results revealed lower *T. semipenetrans* populations in Citromelo and Poncirus, confirming their resistance. Furthermore, the biocontrol potential of *Bacillus* and *Pseudomonas* strains was demonstrated on the susceptible Sour orange. While the precise mechanisms of the bacterial strains in suppressing *T. semipenetrans* warrant further investigation under field conditions, this study provides the critical components for an integrated approach. This parallel evaluation establishes a robust foundation for integrating these core components. Our findings demonstrate their high potential for inclusion in a comprehensive, field-based integrated pest management strategy.
